# Effectiveness of Cooking Procedures in Reducing Antibiotic Residues in Bivalves

**DOI:** 10.3390/antibiotics13121200

**Published:** 2024-12-09

**Authors:** Hugo Bastos, André M. P. T. Pereira, Angelina Pena, Andreia Freitas, Marta Leite, Liliana J. G. Silva

**Affiliations:** 1 LAQV, REQUIMTE, Laboratory of Bromatology and Pharmacognosy, Faculty of Pharmacy, University of Coimbra, Polo III, Azinhaga de Sta Comba, 3000-548 Coimbra, Portugal; hugolopes.bio@gmail.com (H.B.); andrepereira@ff.uc.pt (A.M.P.T.P.); apena@ci.uc.pt (A.P.); 2 National Institute for Agricultural and Veterinary Research (INIAV), I.P., Av. da República, Quinta do Marquês, 2780-157 Oeiras, Portugal; andreia.freitas@iniav.pt (A.F.); martasofia.22@gmail.com (M.L.); 3 Associated Laboratory for Green Chemistry of the Network of Chemistry and Technology, LAQV, REQUIMTE, R.D. Manuel II, 4051-401 Porto, Portugal

**Keywords:** bivalves, antibiotics, cooking procedures, antibiotics removal, food safety, UHPLC-ToF-MS

## Abstract

**Background/Objectives:** The widespread use of antibiotics, which wastewater treatment plants (WWTPs) cannot fully remove, in human and veterinary medicine leads to their release into wastewater, resulting in the contamination of aquatic environments. Bivalves can accumulate these antibiotics, posing a risk to shellfish consumers, including potential antimicrobial resistance. This study aimed to assess how three cooking methods—marinating, steaming, and grilling—affect the concentration of 33 different antibiotics in bivalves fortified at the level of maximum residue limit (MRL) and twice the MRL (2MRL). **Results:** The data show the percentage of antibiotic remaining after cooking: 100% indicates stability or no reduction; values above 100% show an increase in concentration, and values below 100% reflect a decrease in antibiotic concentration. In general, all culinary procedures removed part of the added antibiotics. However, the most effective method was marinating (47%), followed by steaming (60%) and finally grilling (92%). It was also found that, overall, the fortification level, MRL or 2MRL, did not impact antibiotic removal in each cooking method. Moreover, different antibiotics’ classes presented diverse removals when cooked, ranging between 0% for penicillins and 73% for sulphonamides. Furthermore, the results showed a great diversity of responses to cooking within some antibiotic classes. **Methods:** After cooking, the analysis was based on solid–liquid extraction followed by liquid chromatography–quadrupole time-of-flight mass spectrometry (UHPLC-ToF-MS). **Conclusions:** The ongoing monitoring of antibiotic levels is essential, and further research is needed to understand how cooking affects these substances and their metabolites. This will help assess the real risk to consumers and guide risk-mitigation measures.

## 1. Introduction

The use of pharmaceuticals in human and veterinary medicine has increased significantly in recent decades due to various factors, including exponential population growth, medicine advancements and rise in average life expectancy. The use of these substances has not only increased in quantity but also in the diversity of the medications being administered [1].

One of the most significant scientific achievements of the past century was undoubtedly the discovery of antibiotics, which transformed both human and veterinary medicine [2]. The global use of antibiotics has been steadily increasing due to two key factors: the growth of the human population coupled with widespread access to pharmaceuticals, and the rising demand for animal protein, which leads to intensified livestock production and greater antibiotic use [3]. Since 2006, the use of antibiotics as growth promoters has been banned in the European Union. However, in other places worldwide, this practice is still allowed [4].

Approximately 70 to 80% of antibiotics enter wastewater systems in their original form. The main sources of these antibiotics are domestic effluents (around 75% both in Europe and the USA) and hospital effluents (5% and 20%, respectively) that end up in wastewater treatment plants (WWTPs) [3]. Here, antibiotics and their metabolites may undergo biodegradation, adsorption by sludge or they may not undergo any changes, ultimately leading to their presence in the effluent [5].

The treatment methods currently applied do not seem to be completely effective in removing these pharmaceuticals. Previous studies have shown that the removal rate of erythromycin was below 5%, and the removal rates for tetracyclines, sulphonamides, and fluoroquinolones ranged between 30% and 80%. As a result, antibiotics end up being transferred to adjacent water bodies since WWTPs are considered to be the main source of antibiotic contamination in surface waters [3].

When introduced into aquatic environments, pharmaceuticals, with biological activity, can exert toxic effects on living organisms. This occurs primarily through their interactions with cellular structures, disruption of biochemical pathways, and interference with regulatory processes. Additionally, antibiotics’ presence, mainly at concentrations below the inhibitory concentrations, can promote the emergence of bacterial resistance in the aquatic biota [6,7]. Beyond environmental health, human health may also be at risk due to the continuous consumption of contaminated water [8] or from consuming contaminated aquatic organisms.

Bivalves are a class of organisms with a wide geographical distribution, found in saltwater, freshwater, and brackish environments, frequently impacted by anthropogenic activities [9]. Being filtering animals that are widely consumed worldwide, they can accumulate and transfer contaminants, such as antibiotics, through the food chain, potentially threatening human health [10]. The risk to consumers can manifest either directly or indirectly, including the potential for antimicrobial resistance mediated by these pharmaceutical compounds [11].

In addition to their socio-economic and cultural significance, the consumption of bivalves also provides substantial health benefits. These organisms are excellent sources of essential nutrients, offering high-quality protein, minimal saturated fatty acids, and micronutrients, including vitamins. Additionally, the inclusion of bivalves in diets has been associated with improved cognitive development, enhanced vision, and a reduced risk of cardiovascular diseases [12].

Nonetheless, the presence of antibiotics in bivalves was already reported. Ronidazole, sulfamethoxazole, and azithromycin were detected in three bivalve species from the Ebro River delta, in concentrations of up to 3.0 ± 0.1 µg/kg for azithromycin in *Crassostrea gigas* [13]. Azithromycin, along with dimetridazole, sulfamethoxazole, and ronidazole, was also detected in bivalves, with azithromycin found in all analyzed samples (*n* = 50). Its concentration ranged from 1.3 ng/g dw in clams (*Chamelea gallina*, Ebro Delta) to 13.3 µg/kg dw in mussels (*Mytilus galloprovincialis*, Po Delta). Similar high levels were also observed in mussels from the Tagus Estuary (11.8 µg/kg dw) [14]. Another study across various FAO regions reported antibiotic levels in bivalves from Spain and the North Adriatic Sea in concentrations ranging from 0.55 µg/kg of tetracycline in mussels from Atlantic Spain to 125.03 µg/kg of oxytetracycline in clams from the North Adriatic Sea. The latter exceeded the European Union’s maximum residue limit (MRL) for fish [15].

Therefore, it is evident that besides investigating the potential risks resulting from the bioaccumulation of antibiotics by these aquatic organisms, it is essential to assess the impact of culinary treatments on these compounds. Knowing that each antibiotic family has different physicochemical properties, such as water solubility, octanol/water partition coefficient (log Kow), and dissociation constants (pKa) that can lead to different behaviors [5], assessing whether they increase or decrease to negligible levels, when seafood, particularly bivalves, is cooked is of utmost importance [11].

The risk of human exposure to pharmaceutical residues through the food chain is real. However, there is a lack of studies on the effective exposure of these compounds, making it difficult to accurately evaluate the current risk of exposure [11]. Studies describing the effect of cooking processes on the residue levels of pharmaceuticals, namely antibiotics, in seafood are scarce [11,16,17,18,19] and differ in terms of the experimental design, particularly, in terms of the cooking procedures attempted. Our aim was to evaluate the effects of three cooking procedures, namely marinating, steaming, and grilling, on the concentrations of 33 antibiotics, normally controlled in monitoring and surveillance plans for food of animal origin, belonging to different groups, in bivalves fortified at different levels.

It is also worth mentioning that the antibiotics analyzed in this method correspond to those normally controlled in monitoring and surveillance plans for food of animal origin. The maximum residue limit (MRL) established by the European Commission for pharmacologically active compounds that may occur, at residual levels in food of animal origin, is set in the Commission Regulation 37/2010 [20]. In the present study, bivalves were spiked at MRL and twice the MRL (2MRL), followed by solid–liquid extraction and quantification through liquid chromatography coupled with time-of-flight mass spectrometry (UHPLC-ToF-MS).

This study fills a critical gap in understanding how cooking affects antibiotic residues in bivalves, providing valuable insights for food safety regulators, public health officials, and consumers. It also lays the groundwork for future research into cooking-induced chemical transformations and their implications for human health.

## 2. Results and Discussion

### 2.1. Comparison of Cooking Procedures and Fortification Levels

**Figure 1:**
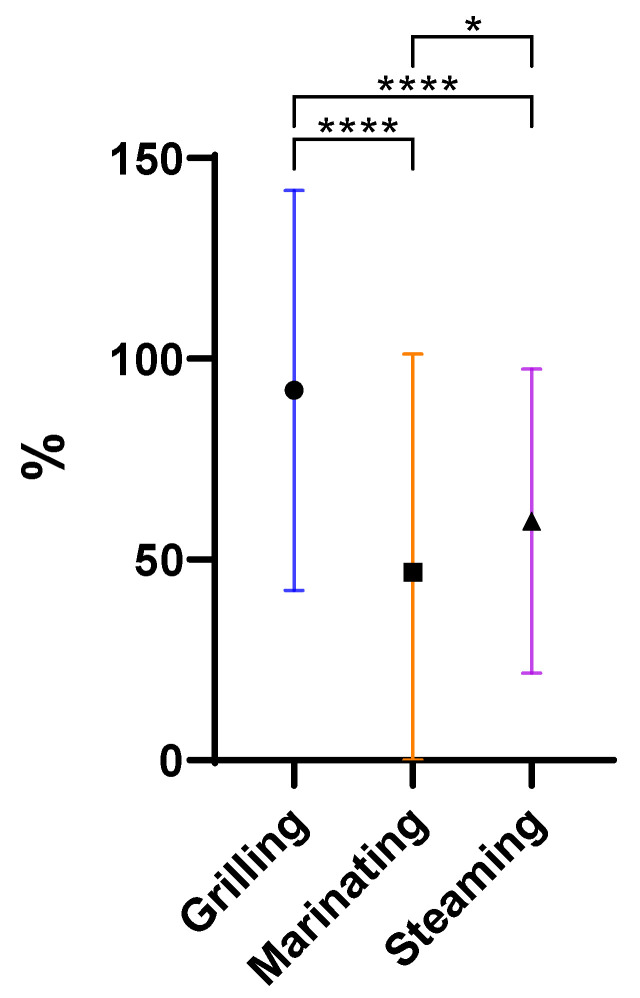
Overall removal (%) of antibiotics when comparing the three cooking procedures under study (**** *p* < 0.0001, * *p* = 0.01 to 0.05).

In general, all culinary procedures partially reduced the antibiotic concentration (Figure 1).

Antibiotics’ degradation, including hydrolysis and epimerization, are increased by high temperatures [21]. Different cooking methods presented different impacts on antibiotic concentrations with statistically significant differences. As seen in Figure 1, there are large statistically significant differences between grilling and the other two cooking procedures and a significant difference between marinating and steaming. However, the most effective removal method was marinating, then steaming and finally grilling. Grilling, with a mean of 92%, was the method that removed the least antibiotics, despite the high temperature provided in this cooking method. Steaming (60%) was more effective at removing antibiotics than grilling, but less effective than marinating (47%). It is known that steam cooking helps to preserve nutrients that are sensitive to heat and water, maintaining a greater integrity of food composition.

**Figure 2:**
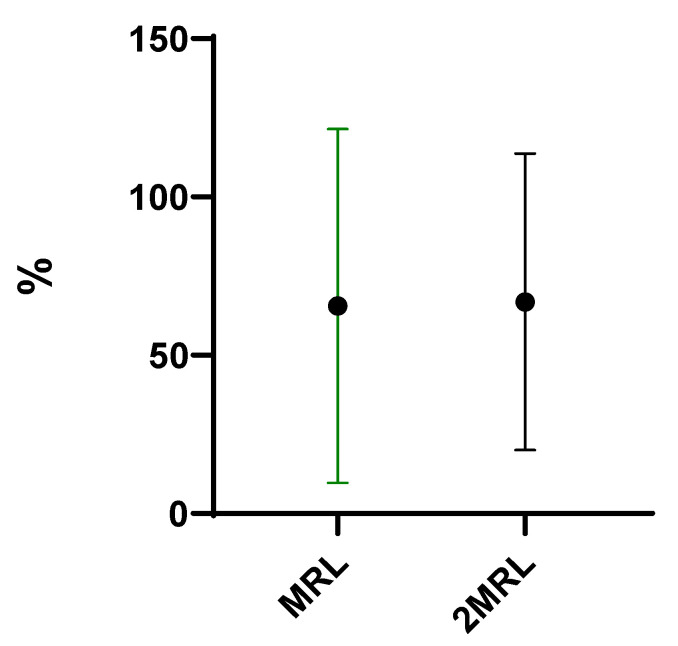
Overall removal (%) of antibiotics when comparing the fortification level (*p* ≥ 0.05).

Finally, marinating was the most effective method for antibiotics removal (47%). This is explained by the fact that the mussels were in direct contact with the aqueous medium and the heating source, which facilitated their biodegradation. On the other hand, the fact that marinating takes place in an acidic conditions, since a part of the marinade’s composition is lemon juice/white wine, also favored the removal of these compounds [21]. These data underline that the grilling of bivalves can increase the risk for consumers, since the weight loss is not compensated with the degradation promoted by this cooking procedure. No statistically significant differences were observed regarding the effect of the two fortification levels, MRL (mean of 67%) or 2MRL (67%), on antibiotic removal (Figure 2).

### 2.2. Comparison Between Antibiotic Groups

According to the results obtained, one can observe that different antibiotic families may behave differently with regards to the effect that cooking has on drug concentration. This was expected given that the different classes of antibiotics have distinct physicochemical characteristics [5].

**Figure 3:**
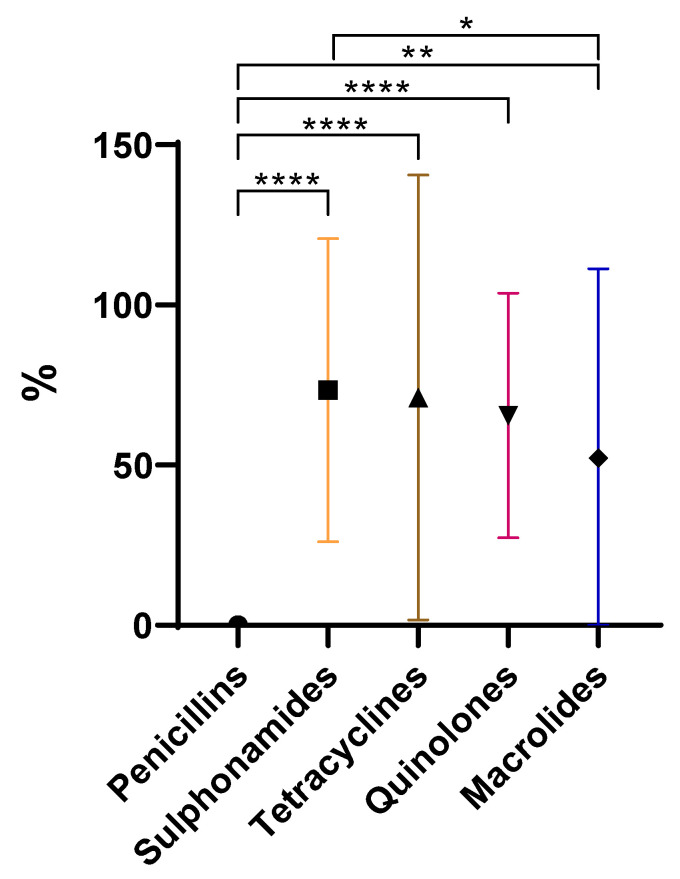
Overall removal (%) of antibiotic groups under study (**** *p* < 0.0001, ** *p* = 0.001 to 0.01, * *p* = 0.01 to 0.05).

In general, all families showed a reduction in antibiotic concentration, which aligns with what has already been stated. As evidenced in Figure 3, there are extremely significant differences between penicillins (mean of 0%) and three other families: sulphonamides (73%), tetracyclines (71%), and quinolones (62%). Additionally, penicillins and macrolides (52%) also exhibit very significant differences.

As expected, it is possible to perceive (Figure 3) that penicillins are the most susceptible to removal during cooking. Penicillins have a beta-lactam ring in their structure, which is prone to degradation [5]. Beta-lactam antibiotics, such as penicillins, have demonstrated a resistance to highly variable temperature increases resulting in the degradation of antibiotics between 20% and 60% [22]. These values appear much lower compared to the 100% degradation observed with cooking methods. However, this discrepancy can be explained by the fact that in cooking, temperature is just one of several factors contributing to the degradation of antibiotics. In the same study, tetracyclines were shown to have highly variable resistance to temperature increases, ranging from less than 20% to more than 50%. The significant variability within these two families of antibiotics has led the authors to conclude that predicting thermal stability based solely on the class of antibiotics is not accurate [22]. Penicillin G, a penicillin and the sole representative of this family in this study, is sensitive to gastric acid due to its low pH [23]. Therefore, we can affirm that the sensitivity of penicillin G to low pH environments, such as those present in marinades, supports the findings that this family is the most sensitive to cooking methods.

There are significant differences between macrolides and sulphonamides. It is noticeable that macrolides are more easily removed than sulfonamides (Figure 3). The difficulty in removing sulphonamides through cooking was expected, as previous studies have shown that sulphonamides can be considered thermotolerant, with very little degradation, around 4%, observed at high temperatures (100–121 °C) [22]. In contrast, macrolides appear to have a lower resistance to temperature compared to sulphonamides. For example, tylosin, a macrolide, in aqueous solution or milk, is removed at a maximum of about 11% at 62 °C for 30 min, 13.31% at 78 °C for 30 s, and 6.47% at 128 °C for 4 s [24]. This is far from the maximum 4% degradation observed in sulphonamides. This fact helps explain why sulphonamides are more resistant to degradation than macrolides when subjected to cooking procedures.

#### 2.2.1. Penicillin

The only representative of the penicillin family in this study is penicillin G, also known as benzylpenicillin. The results show that all three cooking methods completely eliminated this antibiotic in the samples (Figure 3, Table S2), with this being the most susceptible family. Penicillin G is sensitive to temperature and its degradation, by up to 60%, can occur when subjected to high temperatures [22].

On the other hand, this antibiotic is also sensitive to acidic pH, such as that found in marinades, since the bicyclic ring system, which consists of a four-membered ring and a five-membered ring, causes penicillin G to have a large torsion angle. The acidic medium has a catalytic effect on the rupturing and consequent opening of the four-membered ring, leaving it with less tension [23]. We can conclude that penicillins are more susceptible to degradation mainly because of the beta-lactam ring in their structure [5]. Therefore, these results are in line with what was expected; however, to obtain a broader representation of this family, other antibiotics should be studied in the future.

#### 2.2.2. Macrolides

**Figure 4:**
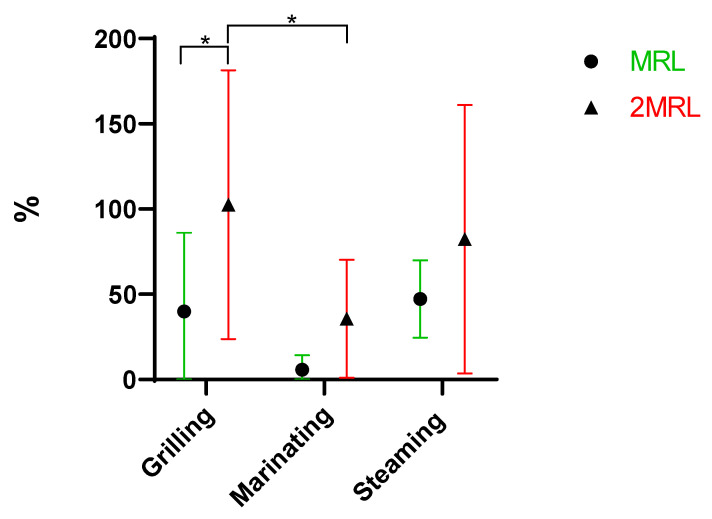
Removal (%) of macrolides when comparing the three cooking procedures under study and fortification levels (* *p* = 0.01 to 0.05).

Macrolides are a class of antibiotics characterized by a large and robust macrocyclic lactone ring, which contributes to their moderate heat stability and limited water solubility properties [25], influencing their behavior during cooking procedures. For the macrolide family, the results indicate that there are statistically significant differences between the fortifications for the grilling cooking method (Figure 4), with a greater removal achievement in the samples subject to MRL fortification. A similar result was found by other authors for two other antibiotics from the macrolide family: clarithromycin and roxithromycin. By increasing the concentration of these drugs, their degradation through photooxidation decreased. These authors explained that at higher concentrations, the solution would be less transparent to UV light, and part of this light would be absorbed by the antibiotic molecules, resulting in a decrease in light reaching the catalyst, thus reducing the formation of OH radicals [25]. A similar phenomenon may explain the fact that, in this specific case, there were significant differences between the two fortifications for grilling.

There were statistically significant differences between grilling (mean of 103%) and marinating (36%) for the 2MRL fortification (Figure 4). When comparing marinating and grilling, the first was the most successful procedure for removing the antibiotics in this family. This was expected since marinating provides an acidic pH with high temperatures conducive to the degradation of most macrolides [24,26], while the grilling method only provides heat. Additionally, grilling was also the method that resulted in the greatest loss of water and, consequently, volume, leading to a higher concentration of macrolides.

#### 2.2.3. Tetracyclines

**Figure 5:**
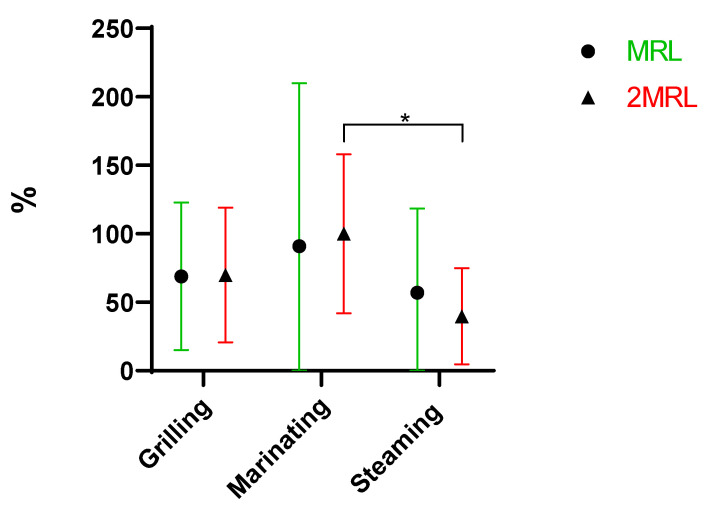
Removal (%) of tetracyclines when comparing the three cooking procedures under study and fortification levels (* *p* = 0.01 to 0.05).

Tetracyclines have a four-ring molecular structure that influences their stability and behavior. As expected, in the tetracycline family, there were no statistically significant differences between both fortification levels (Figure 5) since some authors reported that the effect of high temperatures, a characteristic of every cooking method applied, does not depend on the concentration of tetracyclines [22].

In 2MRL fortification, there are statistically significant differences between marinating (mean of 100%) and steam cooking (40%) (Figure 5). The marinade appears to be less effective at removing antibiotics from the tetracycline family than steam. This could be explained by the fact that, contrary to other antibiotics, tetracyclines are relatively stable in acidic environments, but not in basic pH [27]. Therefore, the acidic pH of marinades was not a relevant factor in the removal of tetracyclines. The removal of tetracyclines was due to the steam generated in the steam cooking since, as was already reported, heated water exhibits identical characteristics to organic solvents [11] and this phenomenon is responsible for the greater removal of tetracyclines.

#### 2.2.4. Quinolones

**Figure 6:**
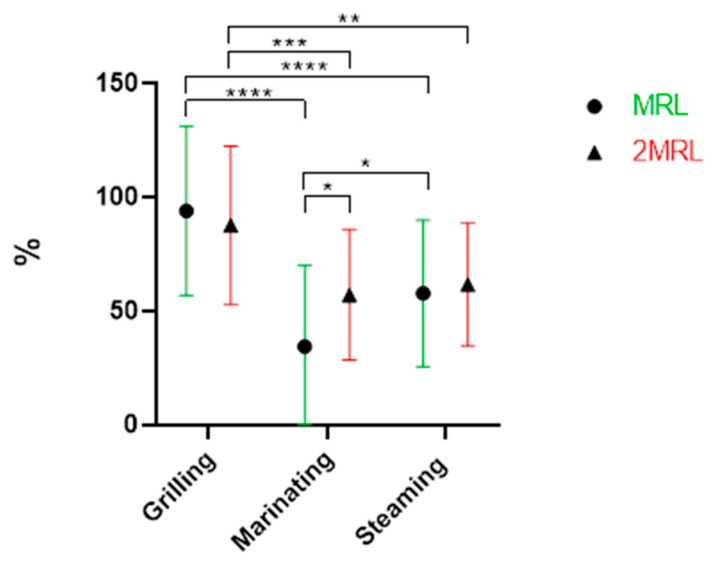
Removal (%) of quinolones when comparing the three cooking procedures under study and fortification levels (**** *p* < 0.0001, *** *p* = 0.0001 to 0.001, ** *p* = 0.001 to 0.01, * *p* = 0.01 to 0.05).

The chemical structure of quinolones is characterized by a bicyclic core structure with a quinoline ring and a carboxylic acid group [28], generally heat-stable, moderately hydrophilic and pH sensitive. In the quinolone family, it was possible to observe a statistically significant difference between MRL and 2MRL fortifications for marinade cooking (Figure 6). Fortification at the MRL favored degradation more than fortification at 2MRL. The results from another study showed that acidic pH conditions combined with low initial concentrations of ciprofloxacin (quinolone used in the study) promoted its removal through γ radiation [29].

In MRL fortifications, there were extremely statistically significant differences between grilling (mean of 94%) and marinating (34%) and between grilling and steaming (58%) (Figure 6). Marinades and steam were more efficient in removing drugs from this family than the grilling method. This was expected considering that high temperatures [28] and acidic pH conditions [30], as provided by the marinade, seem to help in the degradation of quinolones. In the case of steam, the combination of water and a high temperature [11] allowed for greater degradation than in grilling, that only provided a high temperature. At this fortification level, there were statistically significant differences between the marinade and steam (Figure 6). According to the results, the marinade promoted a greater removal of quinolones than steam. This was also expected, since ciprofloxacin, which is a quinolone, is more easily degraded in acidic pH [30].

In 2MRL fortification, there were extremely statistically significant differences between grilling (mean of 89%) and marinating (57%) and very significant differences between grilling and steaming (62%) (Figure 6). These results were expected and are supported by what has been mentioned for the MRL fortifications. and show, once again, the greater effectiveness of the steaming and marinating procedures.

#### 2.2.5. Sulphonamides

**Figure 7:**
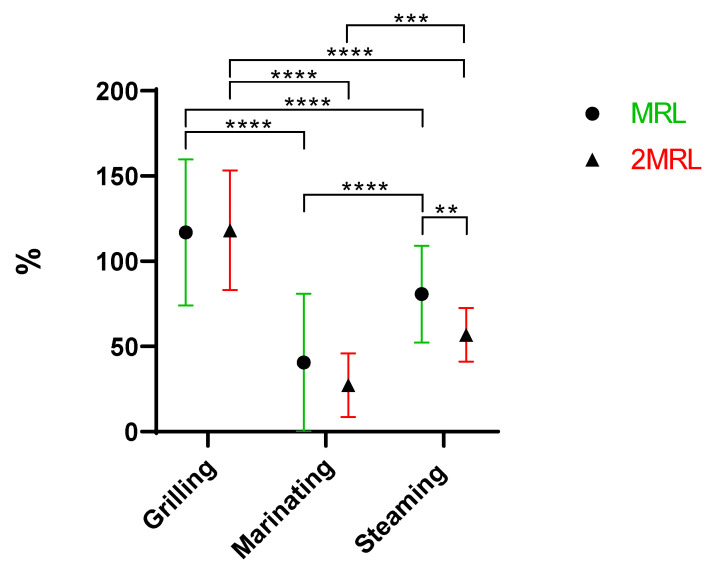
Removal (%) of sulphonamides when comparing the three cooking procedures under study and fortification levels (**** *p* < 0.0001, *** *p* = 0.0001 to 0.001, ** *p* = 0.001 to 0.01).

Sulphonamides present a sulphonamide group (-SO2-NH_2_) attached to an aromatic ring, and is moderately heat-stable, relatively hydrophilic and affected by pH changes [22]. As for the sulphonamide family, the results showed very statistically significant differences between the MRL and 2MRL fortifications for steam cooking (mean of 81% and 57%, respectively) (Figure 7). This is justified by the fact that the degradation of sulphonamides occurs more quickly in water with a higher concentration of these antibiotics [31].

In the samples that underwent MRL and 2MRL fortifications, extremely statistically significant differences were observed between grilling cooking (mean of 117% and 118%, respectively) and the other two methods and between marinating (mean of 41% and 27%, respectively) and steaming (mean of 81% and 57%, respectively) (Figure 7). As mentioned, sulphonamides are considered to be thermotolerant, meaning that little degradation occurs due to high temperatures [22]. The almost non-existent degradation of sulphonamides, together with some water loss, during the cooking procedure caused a small increase in the concentration of this drug in about 75% of the samples in both fortifications. The other two procedures caused a reduction in the concentration of these antibiotics in most samples, with marinating being the most efficient. This result was expected since the acidic environment of the marinade promotes the degradation of most sulphonamide antibiotics [32]. The marinade also promoted a smaller reduction in sample volume. These facts explain the efficiency of marinades in removing sulphonamides.

In this antibiotic family, it is clear that the impact of grilling increases its concentration in the bivalves (17% at the MRL and 18% at 2MRL). This occurs due to its thermotolerance and sample weight loss. Therefore, in this case, it is possible that bivalves with concentrations under the MRL, after grilling, can surpass this value.

Visualizing and interpreting data from antibiotic families is important as it gives us an overview of how each group interacts with cooking methods. However, one should bear in mind that within each family, there is a great variability of physicochemical properties and, therefore, a great diversity of responses. This is easily observed when analyzing the data for each antibiotic individually and comparing them with the data grouped by family. There are some antibiotics with very different cooking behaviors when compared to their family. This is the case of tylosin A (Figure S1), belonging to macrolides, doxycycline (Figure S2), belonging to tetracyclines, cinoxacin (Figure S3), belonging to quinolones and sulfaquinoxaline (Figure S4), belonging to the sulphonamide family.

### 2.3. Comparison with Other Studies

Studies describing the effect of cooking processes on the residue levels of pharmaceuticals, namely antibiotics, in seafood are scarce. Nonetheless, the potential effects on humans caused by the consumption of bivalves contaminated with pharmaceuticals must be investigated, taking into account that much of the fresh seafood undergoes culinary treatments before being consumed. Our results are consistent with others published in scientific literature. Given that the common factor in all three cooking methods is temperature, it is important to underline the considerable stability of antibiotics, even at different fortification levels.

One of the first studies carried out on fish confirmed that certain veterinary antibiotics are poorly degraded during culinary treatments and migrate between tissues [16]. In other studies, attempts were made to investigate the effects of various culinary procedures on antibiotic residues in crustaceans, and the insufficient removal of residues was also observed (20% and 60% in the muscle and shell, respectively) [11].

Other authors aimed to understand the effects of culinary treatments on the concentrations of drugs, including antibiotics, and endocrine disruptors present in seafood, including bivalves, available on the European Union market, as well as to assess the degree of human exposure through the ingestion of this type of food. The samples were collected in two separate campaigns, one in the autumn of 2014 and the other in the spring of 2015, which the authors refer to as round 1 and round 2, respectively. The results revealed that the samples that underwent steam cooking had drug concentrations below the method detection levels (MDL) or below the method quantification levels (MQL) [17]. Therefore, the authors concluded that, regarding the ingestion of cooked seafood products marketed in the European Union in relation to these contaminants, there is no potential risk to human health [17].

On the other hand, another study attempted to understand the effect of cooking on the levels of pharmaceuticals in mussels exposed to concentrations much higher than those detected in nature (around 1000 times higher than in marine surface waters). With the exception of trimethoprim, steaming led to a generalized increase in pharmaceutical residues in both the contaminated mussels’ tissue and in the cooking water. Regarding trimethoprim, a reduction of 23% was observed after steaming [11].

Accordingly, it was also found that oxytetracycline (OTC) and oxalinic acid (OA) levels were reduced in shrimp muscle after culinary treatments. Frying and boiling were the most effective treatments in OTC reduction, and it was concluded that a longer boiling period contributed to a greater reduction up to 80% in the muscle. However, in the shell, the reduction only reached 20%. However, after prolonged treatment, the shrimp muscle became tough and inedible [18]. Regarding OA, a general reduction was recorded in all culinary treatments, between 20 and 30%, in both muscle and shell. Moreover, a longer boiling period contributed to a greater OA reduction of up to 49% [19]. Both studies observed a considerable reduction in both antibiotic residues in this food product; however, they showed that common culinary treatments are unable to completely eliminate these substances. Furthermore, OA residues were relatively thermostable in the shrimp tissues and Ca-tetracycline chelates were thermostable in the shrimp shell [18,19].

## 3. Materials and Methods

### 3.1. Reagents and Solutions

The analytical standards for the 33 targeted antibiotics, with a purity of ≥98%, were purchased from Sigma Chemicals Co. (St. Louis, MO, USA). HPLC grade acetonitrile and methanol were also purchased from Sigma Chemicals Co. (St. Louis, MO, USA). EDTA disodium salt dihydrate (with 99–101% purity) was obtained from Honeywell-Riedel-De Haën (Seelze, Germany), while n-hexane was acquired from Carlo Erba Reagenti (Milan, Italy). Formic acid was purchased from Merck (Darmstadt, Germany) and bi-distilled water was daily obtained through a Milli-Q system (Millipore, Bedford, MA, USA).

Standard stock solutions, including the internal standard (IS), were prepared at a concentration of 1 mg mL^−1^ in methanol, except for penicillin and cephalosporins, which were prepared in water for stability reasons. These stock solutions were stored at −20 °C for 6 months, and appropriate dilutions were made to obtain a final working solution mixture to be used for calibration. The same procedure was followed for the preparation of the sulfameter, the internal standard (IS) working solution, with a concentration of 10 µg mL^−1^.

### 3.2. Experimental Design

Whole mussels (without valves), *Mytilus* spp., weighting 2.0 ± 0.05 g, available for human consumption, were commercially purchased. These mussels originated from Chile and were produced through aquaculture. Table 1 presents the experimental design carried out in a total of 36 assays.

For each cooking process, grilling, marinating (made of white wine, lemon, bay leaf and garlic) and steaming the procedure was as follows: 50 μL of the beta-lactams standard solution was injected, as well as 50 μL of the remaining antibiotics groups, to achieve a fortifying level equivalent to the maximum residue level (MRL). The same procedure was performed for the fortification at twice the MRL (2MRL), adding 100 μL, respectively. All experimental assays were performed in triplicate as described in Table 1. Blanks of cooked and uncooked samples were also analyzed. All samples were weighed before and after cooking (Table S1).

Standard solutions were inserted into the inhalant and exhalant siphon area. The MRL (Table 2) represents the maximum amount of a certain substance that a food may contain, so as not to jeopardize the health of people and animals. Since no specific MRLs are established for bivalve matrices [20], the MRL used was based on the level set for muscle tissue in all food-producing animals. When no such level is specified, the lowest MRL defined for muscle tissue was applied. Since the analyzed matrix was the entire homogenized bivalve, the muscle tissue MRL was considered the closest equivalent.

### 3.3. Sample Extraction and HPLC-TOF-MS Analysis

The analytical methodology used for the identification and quantification of antibiotics in bivalves was based on a previously fully validated method [33]. Chromatograms of uncooked and cooked samples are provided as Supplementary Material (Figure S5).

Briefly, 2.0 ± 0.05 g of a homogenized sample was added of 20 µL of the internal standard solution. The sample was vortexed and allowed to rest for 10 min. Subsequently, 10 mL of acetonitrile and 1 mL of 0.1 M EDTA solution were added and vortexed. The sample underwent homogenization in a vertical shaker for 20 min, following centrifugation at 2879× *g* for 10 min at 4 °C (3–16 K, SIGMA, St. Louis, MO, USA). The resulting supernatant was then transferred to a new centrifugation tube and 2 mL of n-hexane was added; the sample was vortexed and centrifuged under the above referred conditions. The n-hexane phase was discarded, and the remaining acetonitrile phase was evaporated to approximately 0.5 mL.

The chromatographic analysis was conducted on a UHPLC system Shimadzu Nexere X2 coupled with a high-resolution mass spectrometry time-of-flight analyzer (ToF-MS 5600, Sciex, Foster City, CA, USA). A Waters Acquity UPLC HSS T3 1.8 μm, 2.1 × 100 mm chromatographic column (Dublin, Ireland) was used and maintained at a temperature of 40 °C.

For the final extract, an additional 0.5 mL of 0.1% formic acid (mobile phase A) was added and then filtered. Subsequently, 10 µL were injected at a flow rate of 0.5 mL/min, employing a gradient of 0.1% formic acid (A) and acetonitrile (B). Positive electrospray was used, and data acquisition occurred in full-scan mode over a mass range of 100–920 Da. Data acquisition was conducted using the Analyst^®^ TF software version 1.7 (Sciex), and data analysis and results processing were performed utilizing PeakViewTM version 2.2, LibraryViewTM version 1.0.1, and MultiQuantTM version 3.0 (Sciex). Chromatograms of uncooked and cooked samples are provided as Supplementary Material (Figure S5).

### 3.4. Data and Statistical Analysis

This study attempted to investigate the effects of different types of cooking on the concentration of antibiotics in bivalves. The data presented, reflecting the percentage of the amount of the respective antibiotic in relation to its initial pre-cooking amount, was evaluated by means of Equation (1):

(1) $$\%\;Removal=\frac{\left(\frac{Wi}{Wf}\right)\times \;Cf\;}{F}\;\times 100$$

where *Wi* is the initial weight, *Wf* is the final weight, *Cf* is the concentration after cooking, and *F* is the fortification level (MRL or 2MRL). The obtained values were interpreted as follows: a value of 100% denoted high stability/absence of reduction, a value above 100% denoted an enhancement in concentration and a value below 100% denoted a reduction in antibiotic concentration.

Statistical analysis was conducted using GraphPad Prism (version 9, GraphPad Software, Inc., San Diego, CA, USA). To assess the Gaussian distribution of the datasets, the D′Agostino–Pearson normality test was employed. Since none of the datasets exhibited a normal distribution and showed non-homogeneous variances, nonparametric tests were applied. A significance level of *p* < 0.05 was set for all statistical analyses.

The T test was used to assess whether there were significant statistical differences between two independent datasets. A one-way ANOVA was employed to compare three or more independent datasets while a two-way ANOVA was used to evaluate the effect of two independent variables on a variable.

The presented graphics represent the mean and standard deviation.

## 4. Conclusions

In general, all cooking methods promoted a decrease in the concentration of antibiotics in bivalves. However, in most cases, the marinade provided the greatest antibiotic removal, followed by steam cooking and grilling. One should note that it is possible that the antibiotics, when removed from the bivalve through marinade cooking, may migrate to the marinade liquid which, might be also ingested.

Generally, the level of fortification, MRL or 2MRL, did not influence the effect of cooking on antibiotic concentration. However, grouping these pharmaceuticals by class revealed varied behaviors due to the distinct physicochemical properties of each family, highlighting a wide range of responses even within individual classes. The effect of grilling, namely in sulfonamides, increased antibiotic concentration in bivalves, promoting a higher risk.

Therefore, to reduce antibiotic exposure, consumers benefit from using marinades or steaming as preferred culinary methods, whereas regulatory authorities should enhance monitoring programs for antibiotic contamination, namely in aquaculture products.

To protect public health, the continuous monitoring of antibiotic residues in seafood and further investigation into the impact of culinary treatments on these contaminants and their metabolites remain critical. These efforts will support the implementation of evidence-based measures to minimize consumer exposure and ensure food safety.

## Figures and Tables

**Table 1:** Experimental design.

Cooking Process	Grilling	Marinating	Steaming
**Antibiotic levels**	MRL (*n* = 3)	MRL (*n* = 3)	MRL (*n* = 3)
	2MRL (*n* = 3)	2MRL (*n* = 3)	2MRL (*n* = 3)
	Cooked blank (*n* = 3)	Cooked blank (*n* = 3)	Cooked blank (*n* = 3)
	Raw blank (*n* = 3)	Raw blank (*n* = 3)	Raw blank (*n* = 3)

**Table 2:** MRL values (µg/kg) used for each antibiotic in study.

Antibiotic Group	Antibiotic	MRL
**Macrolides**	Tilmicosin	50
	Tylosin A	100
	Trimethoprim	50
**Penicillins**	Benzylpenicillin (pen G)	50
**Quinolones**	Ciprofloxacin	100
	Harmofloxacin	100
	Enrofloxacin	100
	Nalidixic acid	100
	Norfloxacin	100
	Ofloxacin	100
	Enoxacin	100
	Cinoxacin	100
	Marbofloxacin	150
	Flumequina	200
	Oxolinic acid	300
**Sulphonamides**	Sulfachloropyridazine	100
	Sulfadiazine	100
	Sulfadimethoxine	100
	Sulfamethazine	100
	Sulfamethiazole	100
	Sulfapyridine	100
	Sulfaquinoxaline	100
	Sulfathiazole	100
	Sulfisoxazole	100
	Sulfamethoxazole	100
	Sulfisomidine	100
	Sulfadoxine	100
**Tetracyclines**	Chlorotetracycline	100
	Doxycycline	100
	Epi-chlortetracycline	100
	Epi-tetracycline	100
	Oxytetracycline	100
	Tetracycline	100

## Data Availability

The data presented in this study are available upon request from the corresponding author.

## Supplementary Materials

The following supporting information can be downloaded at: https://www.mdpi.com/article/10.3390/antibiotics13121200/s1, Table S1: Weight (g) of each replicate before and after each cooking procedure and fortification level (µg/kg); Table S2: Removal (%) of all antibiotics in the study regarding different fortification levels and cooking procedures; Figure S1: Tylosin A removal percentage; Figure S2: Doxycycline removal percentage; Figure S3: Cinoxacin removal percentage; Figure S4: Sulfaquinoxaline removal percentage; Figure S5: Chromatograms of uncooked and cooked samples.
