# Supplementary material for: Effectiveness of Cooking Procedures in Reducing Antibiotic Residues in Bivalves
Source: Antibiotics (Basel). 2024 Dec 9;13(12):1200. doi: 10.3390/antibiotics13121200 (PMC11672508; doi:10.3390/antibiotics13121200)
Supplement: Supplementary file 1 [file antibiotics-13-01200-s001.zip › antibiotics-3337402-supplementary.pdf]

## Supplementary material

**Table S1.** Weight (g) of each replicate before and after each cooking procedure and fortification level (μg/kg).

| Cooking procedure | Fortification level (μg/kg) | Weight before cooking | Weight after cooking |
|---|---|---|---|
| Marinating | B | 1.98 | 1.707 |
| | B | 2.02 | 1.603 |
| | B | 2.05 | 1.975 |
| | MRL | 1.95 | 1.743 |
| | MRL | 2.05 | 1.758 |
| | MRL | 2.05 | 1.139 |
| | 2MRL | 1.97 | 1.652 |
| | 2MRL | 1.96 | 1.742 |
| | 2MRL | 2.05 | 1.622 |
| Steaming | B | 2 | 1.3 |
| | B | 2.04 | 1.334 |
| | B | 2.02 | 1.486 |
| | MRL | 2.04 | 1.396 |
| | MRL | 1.95 | 1.405 |
| | MRL | 2.04 | 1.669 |
| | 2MRL | 2.04 | 1.52 |
| | 2MRL | 2.04 | 1.421 |
| | 2MRL | 2.056 | 1.5 |
| Grilling | B | 2.04 | 1.347 |
| | B | 1.99 | 1.089 |
| | B | 2 | 1.142 |
| | MRL | 2.01 | 1.405 |
| | MRL | 1.96 | 1.318 |
| | MRL | 2 | 1.1248 |
| | 2MRL | 2 | 1.351 |
| | 2MRL | 2.02 | 1.274 |
| | 2MRL | 1.97 | 1.148 |

**Table S2.** Removal (%) of all antibiotics in study regarding different fortification levels and cooking procedures.

| Group | Antibiotic | Replicate | MRL | | | 2MRL | | |
|---|---|---|---|---|---|---|---|---|
| | | | Grilling | Marinating | Steaming | Grilling | Marinating | Steaming |
| Penicillins | Benzylpenicillin | n=1 | 0 | 0 | 0 | 0 | 0 | 0 |
| | | n=2 | 0 | 0 | 0 | 0 | 0 | 0 |
| | | n=3 | 0 | 0 | 0 | 0 | 0 | 0 |
| Macrolides | Tilmicosin | n=1 | 0 | 0 | 98 | 224 | 115 | 0 |
| | | n=2 | 10 | 0 | 40 | 67 | 54 | 161 |
| | | n=3 | 0 | 0 | 26 | 134 | 0 | 232 |
| | Trimethoprim | n=1 | 82 | 0 | 34 | 201 | 45 | 73 |
| | | n=2 | 81 | 0 | 63 | 102 | 35 | 77 |
| | | n=3 | 130 | 0 | 55 | 139 | 13 | 135 |
| | Tylosin A | n=1 | 18 | 14 | 47 | 13 | 17 | 14 |
| | | n=2 | 26 | 20 | 30 | 6 | 33 | 22 |
| | | n=3 | 12 | 17 | 32 | 37 | 9 | 27 |
| Sulfonamides | Sulfachloropyridazine | n=1 | 109 | 43 | 95 | 102 | 34 | 52 |
| | | n=2 | 124 | 39 | 84 | 114 | 27 | 60 |
| | | n=3 | 150 | 64 | 76 | 94 | 23 | 60 |
| | Sulfadiazine | n=1 | 87 | 0 | 29 | 203 | 0 | 51 |
| | | n=2 | 110 | 0 | 52 | 132 | 0 | 44 |
| | | n=3 | 122 | 0 | 68 | 147 | 0 | 54 |
| | Sulfadimethoxine | n=1 | 110 | 78 | 113 | 76 | 51 | 54 |
| | | n=2 | 123 | 67 | 98 | 113 | 47 | 61 |
| | | n=3 | 158 | 122 | 88 | 80 | 43 | 63 |
| | Sulfadimidine | n=1 | 81 | 0 | 73 | 187 | 10 | 72 |
| | | n=2 | 101 | 0 | 48 | 140 | 0 | 68 |
| | | n=3 | 132 | 0 | 93 | 129 | 0 | 67 |
| | Sulfadoxine | n=1 | 56 | 9 | 72 | 127 | 36 | 36 |
| | | n=2 | 68 | 0 | 49 | 107 | 41 | 50 |

| Group | Antibiotic | Replicate | MRL | | | 2MRL | | |
|---|---|---|---|---|---|---|---|---|
| | | | Grilling | Marinating | Steaming | Grilling | Marinating | Steaming |
| | | n=3 | 97 | 0 | 55 | 88 | 17 | 45 |
| | Sulfamethizole | n=1 | 144 | 51 | 80 | 134 | 37 | 58 |
| | | n=2 | 162 | 42 | 93 | 137 | 36 | 57 |
| | | n=3 | 187 | 69 | 101 | 120 | 26 | 74 |
| | Sulfamethoxazole | n=1 | 89 | 56 | 101 | 100 | 47 | 47 |
| | | n=2 | 105 | 46 | 83 | 107 | 44 | 59 |
| | | n=3 | 132 | 74 | 81 | 81 | 36 | 58 |
| | Sulfapyridine | n=1 | 107 | 0 | 71 | 192 | 5 | 70 |
| | | n=2 | 119 | 0 | 54 | 148 | 15 | 59 |
| | | n=3 | 153 | 0 | 80 | 147 | 0 | 61 |
| | Sulfaquinoxaline | n=1 | 100 | 75 | 101 | 60 | 44 | 48 |
| | | n=2 | 109 | 67 | 90 | 93 | 38 | 51 |
| | | n=3 | 135 | 116 | 80 | 69 | 38 | 55 |
| | Sulfathiazole | n=1 | 186 | 92 | 142 | 153 | 54 | 93 |
| | | n=2 | 197 | 92 | 147 | 131 | 54 | 88 |
| | | n=3 | 234 | 134 | 145 | 146 | 52 | 96 |
| | Sulfisomidine | n=1 | 42 | 0 | 61 | 152 | 3 | 30 |
| | | n=2 | 38 | 0 | 24 | 100 | 17 | 39 |
| | | n=3 | 74 | 0 | 48 | 92 | 0 | 18 |
| | Sulfisoxazole | n=1 | 77 | 43 | 95 | 80 | 37 | 43 |
| | | n=2 | 97 | 37 | 67 | 98 | 38 | 56 |
| | | n=3 | 95 | 42 | 67 | 73 | 31 | 48 |
| **Quinolones** | Cinoxacin | n=1 | 57 | 99 | 74 | 94 | 116 | 30 |
| | | n=2 | 71 | 64 | 69 | 53 | 110 | 48 |
| | | n=3 | 100 | 87 | 53 | 63 | 89 | 57 |
| | Ciprofloxacin | n=1 | 67 | 30 | 25 | 114 | 67 | 32 |
| | | n=2 | 85 | 7 | 52 | 46 | 56 | 37 |

| Group | Antibiotic | Replicate | MRL | | | 2MRL | | |
|---|---|---|---|---|---|---|---|---|
| | | | Grilling | Marinating | Steaming | Grilling | Marinating | Steaming |
| | | n=3 | 115 | 8 | 26 | 58 | 30 | 82 |
| | Danofloxacin | n=1 | 112 | 62 | 88 | 137 | 67 | 53 |
| | | n=2 | 143 | 52 | 117 | 73 | 60 | 62 |
| | | n=3 | 176 | 76 | 95 | 95 | 45 | 95 |
| | Enoxacin | n=1 | 62 | 25 | 17 | 147 | 99 | 47 |
| | | n=2 | 83 | 0 | 59 | 45 | 66 | 49 |
| | | n=3 | 128 | 0 | 8 | 63 | 33 | 122 |
| | Enrofloxacin | n=1 | 12 | 0 | 6 | 159 | 37 | 45 |
| | | n=2 | 36 | 0 | 11 | 37 | 9 | 45 |
| | | n=3 | 51 | 0 | 26 | 70 | 0 | 142 |
| | Flumequine | n=1 | 113 | 94 | 94 | 99 | 89 | 52 |
| | | n=2 | 121 | 73 | 73 | 93 | 86 | 64 |
| | | n=3 | 149 | 120 | 120 | 84 | 70 | 71 |
| | Marbofloxacin | n=1 | 41 | 0 | 28 | 143 | 48 | 39 |
| | | n=2 | 85 | 0 | 30 | 54 | 29 | 37 |
| | | n=3 | 77 | 0 | 15 | 78 | 9 | 99 |
| | Nalidixic Acid | n=1 | 72 | 61 | 86 | 104 | 86 | 38 |
| | | n=2 | 80 | 40 | 75 | 77 | 89 | 57 |
| | | n=3 | 114 | 67 | 69 | 80 | 66 | 56 |
| | Norfloxacin | n=1 | 78 | 18 | 29 | 118 | 59 | 38 |
| | | n=2 | 95 | 0 | 68 | 53 | 42 | 47 |
| | | n=3 | 134 | 3 | 31 | 65 | 29 | 91 |
| | Ofloxacin | n=1 | 110 | 28 | 67 | 176 | 56 | 62 |
| | | n=2 | 126 | 0 | 93 | 75 | 39 | 57 |
| | | n=3 | 160 | 0 | 71 | 110 | 27 | 113 |
| | Oxolinic Acid | n=1 | 63 | 45 | 92 | 93 | 69 | 40 |
| | | n=2 | 75 | 27 | 69 | 66 | 60 | 61 |

| Group | Antibiotic | Replicate | MRL | | | 2MRL | | |
|---|---|---|---|---|---|---|---|---|
| | | | Grilling | Marinating | Steaming | Grilling | Marinating | Steaming |
| | | n=3 | 105 | 50 | 70 | 73 | 48 | 67 |
| **Tetracyclines** | Chlorotetracycline | n=1 | 87 | 22 | 88 | 131 | 53 | 25 |
| | | n=2 | 94 | 6 | 47 | 48 | 44 | 36 |
| | | n=3 | 123 | 41 | 56 | 92 | 31 | 44 |
| | Doxycycline | n=1 | 7 | 167 | 184 | 21 | 109 | 81 |
| | | n=2 | 25 | 144 | 184 | 16 | 89 | 103 |
| | | n=3 | 69 | 293 | 150 | 29 | 94 | 108 |
| | Epi-chlorotetracycline | n=1 | 0 | 33 | 0 | 117 | 97 | 0 |
| | | n=2 | 9 | 0 | 25 | 44 | 92 | 1 |
| | | n=3 | 41 | 0 | 6 | 27 | 66 | 47 |
| | Epi-Tetracycline | n=1 | 134 | 303 | 78 | 133 | 202 | 21 |
| | | n=2 | 175 | 150 | 85 | 77 | 244 | 36 |
| | | n=3 | 147 | 359 | 42 | 80 | 195 | 62 |
| | Oxytetracycline | n=1 | 8 | 0 | 0 | 114 | 115 | 0 |
| | | n=2 | 55 | 0 | 0 | 0 | 71 | 0 |
| | | n=3 | 6 | 0 | 0 | 13 | 77 | 26 |
| | Tetracycline | n=1 | 85 | 34 | 57 | 166 | 97 | 16 |
| | | n=2 | 88 | 0 | 11 | 52 | 82 | 24 |
| | | n=3 | 88 | 84 | 14 | 99 | 42 | 86 |

*antibiotics*

MDPI

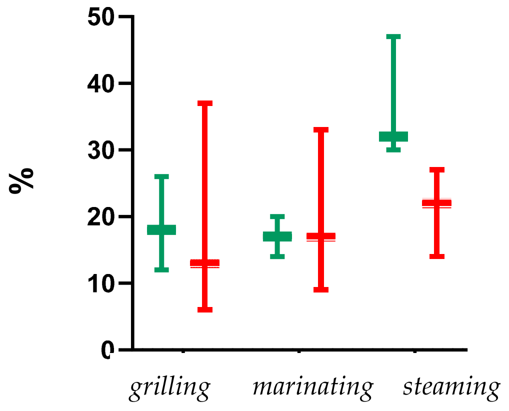


**Figure S1.** Tylosin A removal percentage.

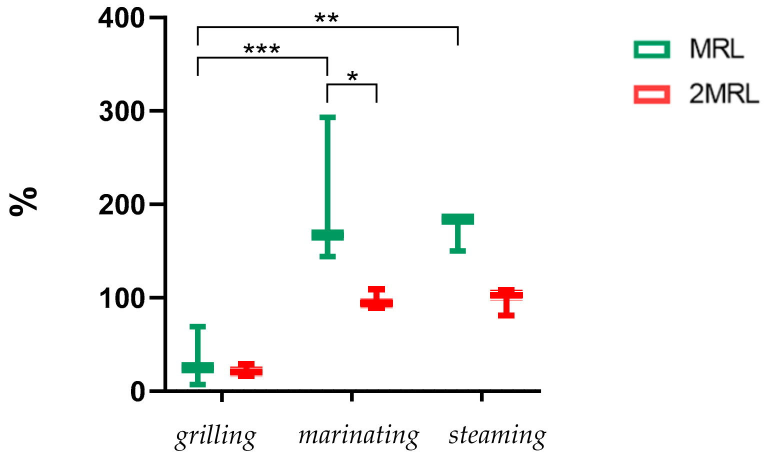


**Figure S2.** Doxycycline removal percentage.

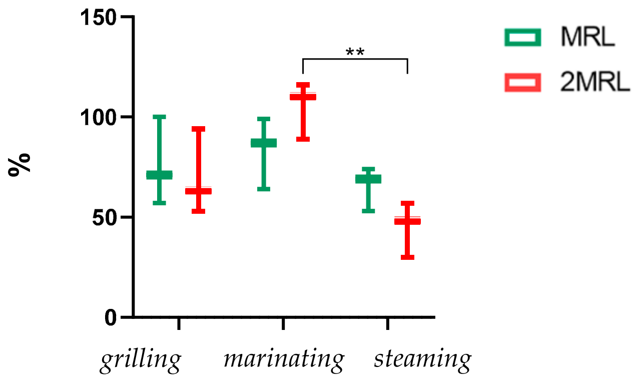


**Figure S3.** Cinoxacin removal percentage.

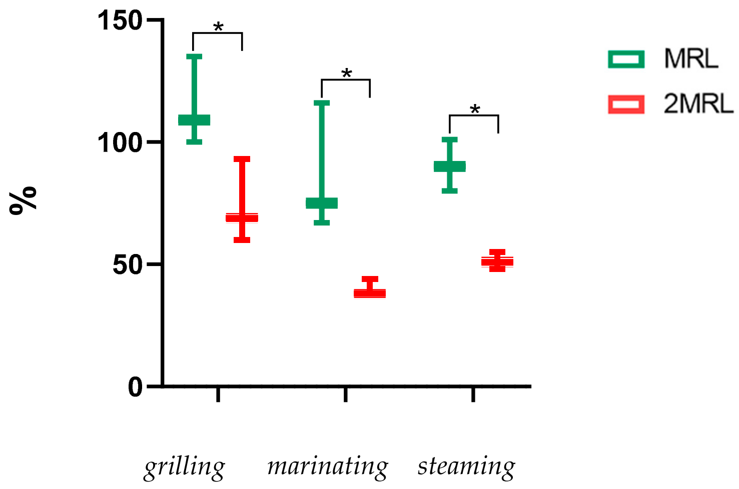


**Figure S4.** Sulfaquinoxaline removal percentage.
